# *In Silico* Prediction of T and B Cell Epitopes of Der f 25 in *Dermatophagoides farinae*

**DOI:** 10.1155/2014/483905

**Published:** 2014-05-08

**Authors:** Xiaohong Li, Hai-Wei Yang, Hao Chen, Jing Wu, Yehai Liu, Ji-Fu Wei

**Affiliations:** ^1^Department of Allergy, First Affiliated Hospital of Anhui Medical University, No. 210, Jixi Road, Anhui Province, Hefei 230022, China; ^2^Department of Urology, First Affiliated Hospital of Nanjing Medical University, Jiangsu Province, Nanjing 210029, China; ^3^Research Division of Clinical Pharmacology, First Affiliated Hospital of Nanjing Medical University, 300 Guangzhou Road, Nanjing 210029, China

## Abstract

The house dust mites are major sources of indoor allergens for humans, which induce asthma, rhinitis, dermatitis, and other allergic diseases. Der f 25 is a triosephosphate isomerase, representing the major allergen identified in *Dermatophagoides farinae*. The objective of this study was to predict the B and T cell epitopes of Der f 25. In the present study, we analyzed the physiochemical properties, function motifs and domains, and structural-based detailed features of Der f 25 and predicted the B cell linear epitopes of Der f 25 by DNAStar protean system, BPAP, and BepiPred 1.0 server and the T cell epitopes by NetMHCIIpan-3.0 and NetMHCII-2.2. As a result, the sequence and structure analysis identified that Der f 25 belongs to the triosephosphate isomerase family and exhibited a triosephosphate isomerase pattern (PS001371). Eight B cell epitopes (11–18, 30–35, 71–77, 99–107, 132–138, 173–187, 193–197, and 211–224) and five T cell epitopes including 26–34, 38–54, 66–74, 142–151, and 239–247 were predicted in this study. These results can be used to benefit allergen immunotherapies and reduce the frequency of mite allergic reactions.

## 1. Introduction

The house dust mites (HDM) are major sources of indoor allergens for humans, which induce asthma, rhinitis, dermatitis, and other allergic diseases [1]. Their major allergens (*Dermatophagoides pteronyssinus* [Der p] and* Dermatophagoides farinae* [Der f]) coexist in most geographical regions with a high proportion (up to 85%) of asthmatics being typically HDM allergic; hence, sensitization is attributed as a risk factor for developing asthma. Recently, a birth cohort study showed that sensitization to HDM at age of 2 years was associated with current wheeze at age of 12 years in both monosensitized and polysensitized HDM-sensitized children [2]. In previous studies, fourteen* D. farinae* allergens (Der f 1–3, 6, 7, 10, 11, 13–18, and 22) were reported before other seventeen allergens belonging to twelve different groups were identified by a procedure of proteomics combined with two-dimensional immunoblotting from* D. farinae *extracts [3, 4]. Among the novel identified* D. farinae *allergens, Der f 25 is a triosephosphate isomerase (TPI) with a molecular weight of 34 kDa, showing 75.6% by immunoblotting and 60% by skin prick positive reaction to dust mite allergic patients, respectively. It represented the major allergen in* D. farinae* [4].

TPI is an enzyme (EC 5.3.1.1) that catalyzes the reversible interconversion of the triose phosphate isomers dihydroxyacetone phosphate and D-glyceraldehyde 3-phosphate [5]. It has been found in nearly every organism searched for the enzyme, including animals such as mammals and insects as well as in fungi, plants, and bacteria. Moreover, some TPIs have been identified as an allergen in fish, midges, crustaceans, and various plants [6–12].

Currently, specific immunotherapy is the only allergen-specific approach for its treatment of mite allergy. The administration of increasing doses of allergen extracts to patients is the method most commonly applied. However, the use of crude extracts has several disadvantages. It could induce severe anaphylactic side reactions or lead to sensitization towards new allergens present in the mixture [13, 14]. Different strategies have been designed to try to overcome these negative effects, as the use of allergen-derived B cell peptides, allergen-derived T cell epitope containing peptides, or vaccination with allergen-encoding DNA [15]. Known epitopes for some of these mite allergens are described in detail in Cui's review [16]. However, there is no report about the epitope of Der f 25 allergen. In the present study, we firstly identified the B and T cell epitopes of Der f 25 allergen by* in silico* approach. It implied their potential utility in a peptide-based vaccine design for mite allergy.

## 2. Methodology

### 2.1. Sequence Retrieval and Phylogenetic Analysis

The complete amino acid sequence of Der f 25 was acquired from the Nucleotide database of NCBI (http://www.ncbi.nlm.nih.gov/) with the accession number of KC305500.1. The amino acid sequence was also used as query to search for homologous sequences through the Swiss-Prot/TrEMBL (Uniprot) (http://www.uniprot.org/) and tBLASTn in NCBI (http://blast.ncbi.nlm.nih.gov/Blast.cgi).

The homologous amino acid sequences were retrieved and aligned using Clustal X 2.1 [17]. Phylogenetic tree was obtained by using ML (maximum-likelihood) method on the basis of the JTT amino acid sequence distance implemented in MEGA 5.1 [18]; the reliability was evaluated by the bootstrap method with 1000 replications.

### 2.2. Domain Architecture Analyses

The possible domains and characteristic motifs and patterns contained in Der f 25 were investigated by Pfam v27.0 (http://pfam.sanger.ac.uk/) [19], Prosite (http://prosite.expasy.org/scanprosite/) [20], InterPRO v46.0 (http://www.ebi.ac.uk/interpro/), and Superfamily v1.75 (http://supfam.cs.bris.ac.uk/SUPERFAMILY/index.html) [21].

### 2.3. Physiochemical Analysis and Posttranslational Patterns and Motifs

Physiochemical analysis including molecular weight, theoretical pI, amino acid composition, instability index, aliphatic index, and grand average of hydropathicity (GRAVY) of Der f 25 was performed by using ProtParam tool (http://web.expasy.org/protparam/). Der f 25 characteristic pattern was checked for original sequence and further analysis was performed to highlight the presence of functional motifs by using the Prosite database (http://prosite.expasy.org/) [20]. Biologically meaningful motifs and susceptibility to posttranslational modifications were derived from multiple alignments and the ScanProsite tool. Phosphorylation motifs with more than 80% of probability of occurrence were analyzed by using NETPhos v2.0 (http://www.cbs.dtu.dk/services/NetPhos/) and NETPhosK v1.0 (http://www.cbs.dtu.dk/services/NetPhosK/) [22].

### 2.4. Secondary Structure Prediction

Der f 25 secondary structural elements were predicted by PSIPRED (http://bioinf.cs.ucl.ac.uk/psipred/) [23], which threads sequence segments through protein data bank (PDB) library (http://www.rcsb.org/) to identify conserved substructures. Furthermore, the secondary structure elements were also identified with the result obtained with NetSurfP ver. 1.1 (http://www.cbs.dtu.dk/) [24].

### 2.5. Homology Modeling and Validation

The Der f 25 protein sequence was searched for homology in the PDB. As well, the homologous templates suitable for Der f 25 were selected by PSI-BLAST server (http://blast.ncbi.nlm.nih.gov/Blast.cgi) and SWISS-MODEL server (http://swissmodel.expasy.org/) [25, 26]. The best template was retrieved from the results of previous methods and used for homology modeling. Der f 25 modeled protein structure was built through alignment mode in SWISS-MODEL using the complete amino acid sequence. An initial structural model was generated and checked for recognition of errors in 3D structure by PROCHECK [27], ERRAT [28], and VERIFY 3D [29] programs in structural analysis and verification server (SAVES) (http://nihserver.mbi.ucla.edu/SAVES/). The final model structure quality of Der f 25 was assessed by QMEAN [30], by checking protein stereology with ProSA program [31] and the protein energy with ANOLEA (http://protein.bio.puc.cl/cardex/servers/anolea/) [32]. The Ramachandran plot for all the models was generated, showing the majority of the protein residues in the favored regions.

### 2.6. Conservation Analysis and Poisson-Boltzmann Electrostatic Potential

Der f 25 model was submitted to ConSurf server (http://consurf.tau.ac.il/) in order to generate evolutionary related conservation scores helping to identify functional regions in the proteins. Functional and structural key residues in Der f 25 sequence were confirmed by ConSeq server [33].

APBS molecular modeling software implemented in PyMOL 0.99 was used to investigate the electrostatic Poisson-Boltzmann (PB) potentials of Der f 25 model structure. AMBER99 in PDB2PQR server (http://nbcr-222.ucsd.edu/pdb2pqr_1.8/) was used to assign the charges and radii to all of the atoms (including hydrogens) [34]. Fine grid spaces of 0.35 Å were used to solve the linearized PB equation in sequential focusing multigrid calculations in a mesh of 130 points per dimension at 310.00 K. The dielectric constants were 2.0 and 80.0 for the protein and water. The output mesh was processed in the scalar OpenDX format to render isocontours and maps onto the surfaces with PyMOL 0.99. Potential values are given in units of *kT* per unit charge (*k* Boltzmann's constant; *T* temperature).

### 2.7. *In Silico* Prediction of B Cell Epitopes

Three immunoinformatics tools including DNAStar protean system, bioinformatics predicted antigenic peptides (BPAP) system (http://imed.med.ucm.es/Tools/antigenic.pl), and BepiPred 1.0 server (http://www.cbs.dtu.dk/services/BepiPred/) were used to predicate the B cell epitopes of Der f 25. The ultimate consensus epitope results were obtained by combining the results of the three tools together with the method published earlier [35]. In the DNAStar protean system, four properties (hydrophilicity, flexibility, accessibility, and antigenicity) of the amino acid sequence were chosen as parameters for epitopes prediction. The BPAP system and the BepiPred 1.0 server only need the amino acid sequence and provide more straightforward results which are combined with physicochemical properties of amino acids such as hydrophilicity, flexibility, accessibility, turns, and exposed surface [36].

### 2.8. *In Silico* Prediction of T Cell Epitopes

T cell epitopes are principally predicted indirectly by identifying the binding of peptide fragments to the MHC complexes. The binding significance of each peptide to the given MHC molecule is based on the estimated strength of binding exhibited by a predicted nested core peptide at a set threshold level. For HLA-DR-based T cell epitope prediction, the artificial neural network-based alignment (NN-align) method NetMHCIIpan-3.0 (http://www.cbs.dtu.dk/services/NetMHCIIpan/) [37] was applied. For HLA-DQ alleles, NetMHCII-2.2 (http://www.cbs.dtu.dk/services/NetMHCII/) [38] was used. In this study, HLA-DR 101, HLA-DR 301, HLA-DR 401, and HLA-DR 501 were used to predict HLA-DR-based T cell epitope prediction. The ultimate HLA-DR-based T cell epitope results were obtained by combining those four results together that if three of them showed epitope, then the consensus result was epitope. This method was also used in HLA-DQ-based T cell epitope prediction. HLA-DQA10101-DQB10501, HLA-DQA10501-DQB10201, HLA-DQA10501-DQB10301, and HLA-DQA10102-DQB10602 were used to predict HLA-DQ-based T cell epitope prediction. As a result, the ultimate consensus epitope results were obtained by combining the results of the HLA-DR-based T cell epitope and HLA-DQ-based T cell epitope.

B cell and T cell epitopes identified by computational tools were mapped onto linear sequence and on the three-dimensional model of Der f 25 to determine their position and secondary structure elements involved.

## 3. Results

### 3.1. Sequence Retrieval and Sequence Analysis

**Figure 1:**
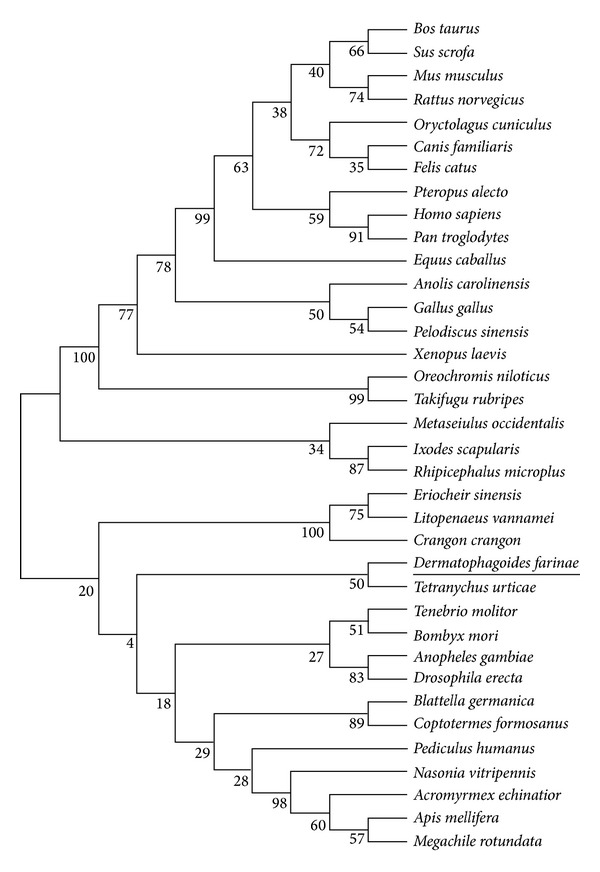
Phylogenetic relationship of Der f 25 amino acid sequence with other homologs.

The amino acid sequence of Der f 25 was obtained from the Nucleotide database of NCBI. Uniprot and tBLASTn were used to search the homologous sequences of Der f 25. As a result, thirty-six sequences were obtained and in order to determine the relationships between Der f 25 and its homologous sequences, phylogenetic analysis was performed and the evolutionary tree inferred by the ML method was showed in Figure 1. Phylogenetic analysis result showed that there are proteins including Der f 25 clustered into the same group, belonging to TIPs. Moreover, domain analysis results showed that Der f 25 belongs to the TIM phosphate binding superfamily (SUPERFAMILY number SSF51351 and InterPro number IPR016040) and TPI family (SUPERFAMILY number SSF51352 and InterPro number IPR000652).

**Figure 2:**
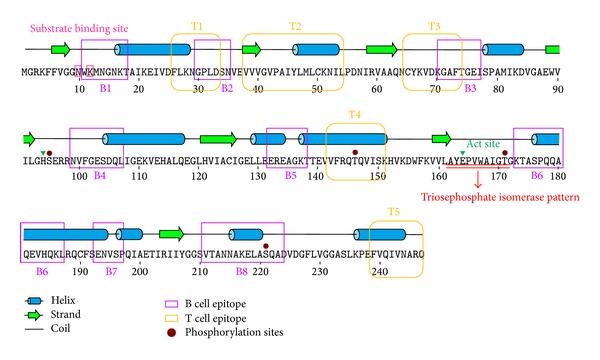
Sequences and second structure analysis of Der f 25 allergen. Ten *α*-helices and seven *β*-sheets were identified in Der f 25.

After searching for characteristic motifs or patterns, we found that Der f 25 exhibited a TPI pattern, PS00171 (162–172, AYEPVWAIGTG) (Figure 2). Phosphorylation sites including two Ser (95 and 221) and two Thr (146 and 171) residues were predicted and showed in Figure 2. Two types of kinases (PKC for 95, 146, and 171 and DNAPK for 221) were predicted to be phosphorylated for Der f 25 complete sequence.

The primary structure of Der f 25 contained 247 amino acids and the molecular weight is 27134.1. The theoretical pI is 6.24 and the aliphatic index is 95.06. The GRAVY is −0.103 meaning that Der f 25 exhibited hydrophilic character. The instability index is 30.57 meaning that the sequence of Der f 25 is stable.

### 3.2. Homology Modeling and Validation

**Figure 3:**
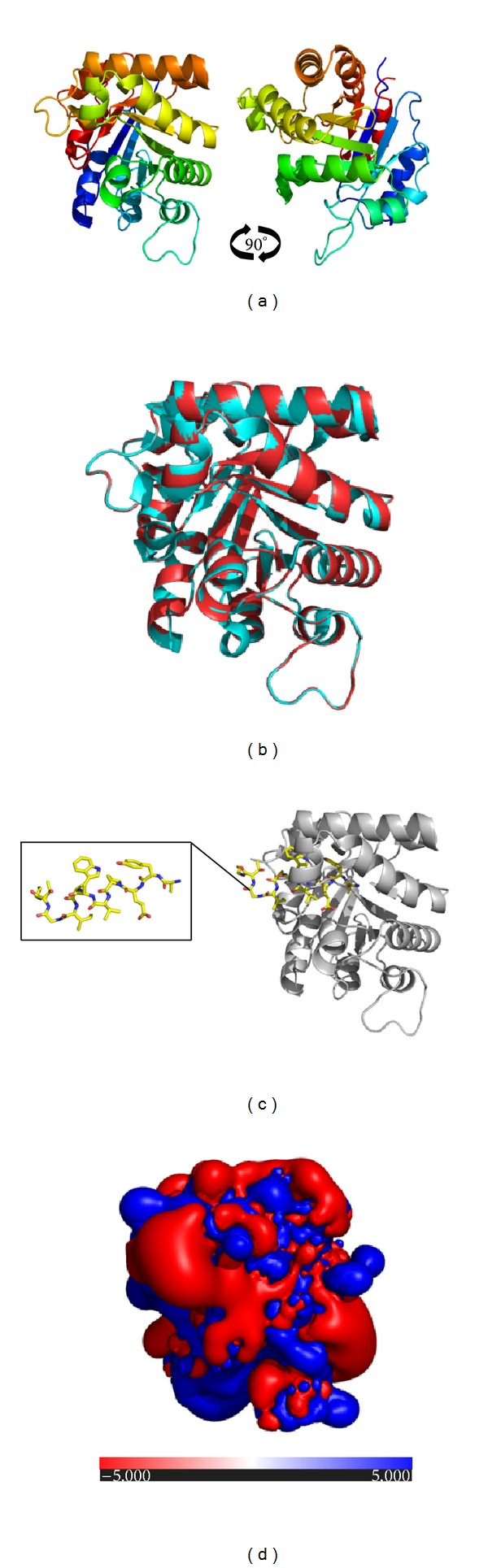
Structural analysis of Der f 25. (a) Protein structure of Der f 25 homology model. (b) Superimposition between Der f 25 and 2I9E template. Der f 25 is depicted in red and 2I9E template is depicted in cyan. (c) Distribution of characteristic pattern in Der f 25. (d) Electrostatic potential representation on the Der f 25 protein surface.

Searching for the proteins with known tertiary structure in the PDB yielded* Tenebrio molitor* TPI (PDB accession number: 2I9E) showing the highest sequence identity (74%) with Der f 25. The SWISS-MODEL server was also used to identify the best possible template and found a high score of 365 and very low *E*-value of e-101 for 2I9E template. Hence, the 2I9E template was used for homology modeling. As indicated by the Ramachandran plot (Figure 3(b)), 93% residues in Der f 25 model were within the most favored regions, 7% residues in the additional allowed region, 0% residues in the generously allowed regions, and 0% residues in the disallowed region; 93.4% residues in 2I9E template were within the most favored regions, 6.6% residues in the additional allowed region, 0% residues in the generously allowed regions, and 0% residues in the disallowed region. The goodness factor (*G*-factor) based on the observed distribution of stereochemical parameters (main chain bond angles, bond length, and phi-psi torsion angles) returned accurate values for a reliable model (Table 1), in comparison with the template 2I9E (Table 1). As indicated by the ERRAT program, the result showed that the overall quality factor is 97.034 for Der f 25 and 97.789 for 2I9E meaning that both of the two structures have good high resolution. As indicated by the VERIFY 3D program, the result showed that 99.6% and 99.8% of Der f 25 and 2I9E template residues had an average 3D (atomic model)-1D (amino acid sequence) score >0.2 also meaning that those two structures were good. ProSa analysis returned *Z*-scores of −10.1 and −10.24 for Der f 25 and 2I9E (Table 1), respectively. *Q* values for Der f 25 and 2I9E structures are 0.772 and 0.798, respectively. Root mean square deviations (RMSD) between Der f 25 structural model and 2I9E template *Cα* backbone are 0.062 Å (Table 1). Based on these validations, it is shown that the homology model was adopted for this study.

### 3.3. Structure Analyses

Secondary structure prediction of Der f 25 with PSIPRED identified ten *α*-helices and seven *β*-sheets (Figure 2) in Der f 25. Alternatively, NetSurfP v1.1 predicted nine *α*-helices and eight *β*-sheets. These results were predicated by different servers and have subtle distinction. The best template 2I9E was used for homology modeling; the overall 3D structure of Der f 25 was shown in Figure 3(a). Sequence polymorphism was responsible for the changes in the spatial distribution of the skeleton alpha carbons, which is reflected in differences between the structures of Der f 25 and 2I9E. A superposition of the Der f 25 with the 2I9E template is shown in Figure 3(b) and the values for superimposed *Cα* are 0.062 Å. As a TPI protein, Der f 25 has two active sites; His in the 94th position is an electrophile while Glu in the 164th position is the proton acceptor. It has two substrate binding sites, the Asn in the 10th position and the Lys in the 12th position. Moreover, the characteristic pattern predicted by ScanProsite tool is shown in Figure 3(c).

### 3.4. Conservational Analysis and Electrostatic Potential

ConSurf conservational analysis of structural and functional key amino acids showed that the Der f 25 protein surfaces were not well conserved, with almost forty high variability residues in different superficial areas. All of the amino acids in the TPI pattern (AYEPVWAIGTG) are conserved. Surface electrostatic potential analysis reveals several prominent charged residues, with half of the side exhibiting large positive values (blue regions) and the other half showing predominantly negative values (red regions) (Figure 3(d)).

### 3.5. B Cell Epitopes Prediction

**Figure 4:**
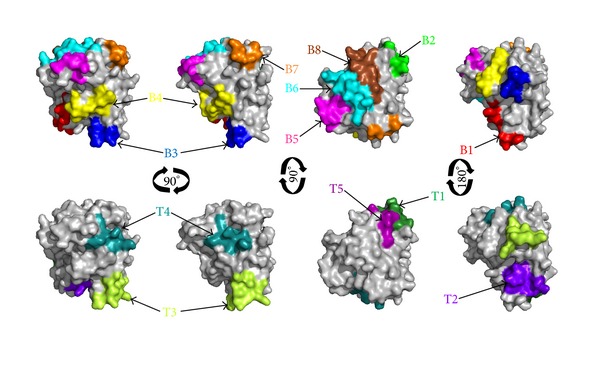
B cell and T cell epitopes superimposition on the surface of Der f 25 allergen structure.

Surface accessibility and fragment flexibility are important features for predicting antigenic epitopes. In addition, the existence of regions with high hydrophobicity also provides strong evidence for epitope identification. Antigenic index directly showed the epitope forming capacity of the Der f 25 sequence. Based on these sequence properties, the final predicting regions of DNAStar were 11–19, 28–35, 68–77, 95–111, 130–139, 173–187, 193–197, and 211–225. Also, the predicted results of the BPAP system were 20–26, 32–52, 54–70, 86–93, 138–168, 175–209, and 218–242 and BepiPred 1.0 server were 11–18, 30–34, 71–78, 99–107, 132–138, 168–183, 194–199, and 210–224. Furthermore, the final potential B cell epitopes of Der f 25 were selected on the basis of the results of these three tools. The ultimate results of the three immunoinformatics tools finally predicted eight peptides (11–18, 30–35, 71–77, 99–107, 132–138, 173–187, 193–197, and 211–224) (Table 2) and these peptides were also shown in Figures 2 and 4.

### 3.6. T Cell Epitopes Prediction

NetMHCIIpan-3.0 and NetMHCII-2.2 were used to identify the T cell epitope of Der f 25. For HLA-DR-based T cell epitope prediction, the final predicting regions of HLA-DR 101, HLA-DR 301, HLA-DR 401, and HLA-DR 501 were shown in Table 1 and the ultimate results of HLA-DR-based T cell epitope prediction finally predicted five peptides (26–34, 38–54, 66–74, 142–151, and 239–247). For HLA-DQ alleles, the final results of HLA-DQA10101-DQB10501, HLA-DQA10501-DQB10201, HLA-DQA10501-DQB10301, and HLA-DQA10102-DQB10602 were also shown in Table 3 and the ultimate results of these four methods finally predicted one peptide, 39–48. As a result, Der f 25 was predicted to have five T cell epitope sequences including 26–34, 38–54, 66–74, 142–151, and 239–247 (Table 2) as shown in Figures 2 and 4.

## 4. Discussion

The prevalence of human atopic disorders including allergic rhinitis, asthma, and atopic dermatitis is increasing during the past several decades. House dust mite allergies constitute more than 50% of allergic patients and often have severe forms of respiratory allergy, such as asthma [1]. Characterization of mite allergens will be beneficial in the diagnosis and treatment of mite-induced atopic illnesses.

Among the identified allergens, Der f 25 is a new protein with a molecular weight of 34 kDa, representing the major allergen in* D. farinae* [4]. The objective of this study was to predict the B and T cell epitopes of Der f 25. Firstly, in order to better understand the structure and function of Der f 25, we analyzed the basic sequence properties and studied the 2D and 3D structures of Der f 25. Phylogenetic analysis result showed that Der f 25 protein clustered into the TIPs group; domain analysis also proved a strong evidence illustrating that Der f 25 belongs to the TPI family. In 2D structure analysis, it is clearly shown that Der f 25 composed of ten *α*-helices and seven *β*-sheets. The 3D structure of Der f 25 was performed by homology modelling which was widely used in many areas of structure-based analysis and study [39]. PDB server was used to search templates of Der f 25 and found that the structure of* Tenebrio molitor* TPI (2I9E) was the best template with the highest identity. Also, small dissimilates in RMSD were observed between Der f 25 and 2I9E template. The built model structure is feasible by the Ramachandran plot analysis, ERRAT program, VERIFY 3D program ProSa analysis, *Q* values, and RMSD. All of these validations showed that the homology model was available. The similar methods for the structures modelling were also successfully conducted in other allergens of Api SI and Api SII [40], Der f 5 [41], Ole e 2 [42] Ole e 11 [39], and Ole e 12 [43]. Based on the conservational analysis of the primary sequence of Der f 25, it is found that almost forty high variability residues sit in different superficial areas of protein surface. The active sites His in 94th position and Glu in 164th position as well as the substrate binding sites Asn in 10th position and Lys in 12th position are the completely conserved sites.

*In silico *prediction has already become a familiar and useful tool for selecting epitopes from immunological relevant proteins, which can save the expense of synthetic peptides and the working time [44]. Recently, many algorithms have been developed to predict B cell epitopes on a protein sequence based on propensity values of amino acid properties of hydrophilicity, antigenicity, segmental mobility, flexibility, and accessibility [45]. In the present study, we used three algorithms (DNAStar protean system, BPAP, and BepiPred 1.0 server) to predict the B cell epitopes. The previous study showed that the use of bioinformatics approach to predict B cell epitopes correlated well with the experimental approach [46]. Earlier study showed that allergen epitopes were comprised of a high proportion of hydrophobic amino acids [47]. The amino acids Ala, Ser, Asn, Gly, and particularly Lys play a key role in the IgE binding allergenic epitopes [48]. In our results, nearly half of the total residues lying in B cell epitopes were hydrophobic (Table 2). Moreover, each predicted B cell epitope has one or more special five amino acids and the common residues in all B cell epitopes were Gly and Lys (Table 2). Electrostatic interactions are known to determine the orientation of the molecules and stabilize antigen-antibody complexes [49]. Surface electrostatic potential analysis result showed that a great part of Der f 25 side exhibits large positive values (blue regions). Most parts of B cell epitopes are distributed in the blue regions and showed a strong negative potential. As a result, eight peptides (11–18, 30–35, 71–77, 99–107, 132–138, 173–187, 193–197, and 211–224) were predicted as the B cell epitopes. However, these B cell epitopes need further investigation in clinical samples.

In the last several years, some methods have substantially improved their accuracy to predict T cell epitopes such as NetMHCpan-3.0 and NetMHCII-2.2. NetMHCpan-3.0 is based on artificial neural networks and is trained on 52,062 quantitative peptide binding data covering all HLA as well as two mouse molecules. In this study, it was used to predict the HLA-DR-based T cell epitopes. For HLA-DQ-based T cell epitopes prediction, NetMHCII-2.2 was used. Although limited binding-affinity data are available for HLA-DQ, it was recently reported to provide the best performance in predicting this locus [50]. As a result, NetMHCIIpan-3.0 and NetMHCII-2.2 were used to predict the T cell epitopes in Der f 25 allergens and predicted 7 potential T cell epitope sequences including 26–34, 38–54, 66–74, 142–151, and 239–247. Despite the high accuracy of these predictions, this approach has not yet been applied to peptide-based vaccine development for allergic diseases.

Allergen-specific immunotherapy (SIT) represents the only allergen-specific and disease-modifying approach with long lasting effects for the treatment of allergic patients [51]. However, SIT can induce side effects, ranging from mild and local to severe and life-threatening symptoms, such as anaphylactic shock [52]. Severe side effects are frequently observed in patients with house dust mite (HDM) allergy [53]. The continuous exposure to HDM allergens further complicates the treatment of patients with HDM allergy. Additionally, the quality of natural HDM allergen extracts and vaccines based on these extracts is often poor. Attenuated allergenic molecules, that is, hypoallergens or synthetic peptide fragments, have been used as high dose and safer alternatives to conventional extract-based SIT [54]. Vaccination with a combination of small peptides that together extend across the entire native allergenic protein theoretically could preserve T cell activation while avoiding IgE-based immune responses. IgE recognizes conformational epitopes of larger peptides (B cell epitopes) and proteins while T cell receptors recognize small linear peptides of 8 to 10 amino acids (T cell epitope). By immunizing with small peptides, T cell activation could occur while IgE binding would be lost [55, 56]. Then, we predicted B and T cell epitopes of Der f 25 allergen, the major allergen in HDM, using* in silico* method which can be used to benefit allergen immunotherapies and reduce the frequency of allergic reactions. However, their accuracies need to be confirmed in the further experiments.

## 5. Conclusion

In this study, we have a better understanding of the 2D and 3D structures of Der f 25 and have predicted eight B cell epitopes (11–18, 30–35, 71–77, 99–107, 132–138, 173–187, 193–197, and 211–224) and five T cell epitope including 26–34, 38–54, 66–74, 142–151, and 239–247 of this TPI. All these results can be used to benefit allergen immunotherapies and reduce the frequency of mite allergic reactions.

## Figures and Tables

**Table 1:** Parameters used for proteins structural assessment.

Protein	Structural assessment methods	Ramachandran plot (%)	*G*-factor	MCBL (%)	CBA (%)	*Z*-score	*Q* value	RMSD
Der f 25	PROCHECK analysis	93.0^E^	0.10^I^	100.0	99.0			
		7.0^F^	0.42^J^					
		0.0^G^	0.23^K^					
		0.0^H^						
	ProSa					−10.1		
	QMEAN						0.772	
	RMSD							0^L^

2I9E	PROCHECK analysis	93.4%^E^	0.25^I^	100.0	98.9			
		6.6%^F^	0.55^J^					
		0.0^G^	0.37^K^					
		0.0^H^						
	ProSa					−10.24		
	QMEAN						0.798	
	RMSD							0.056^L^

MCBL: distribution of the main chain bond lengths; CBA: distribution of the covalent bond angles.

^E^Residues in favorable regions; ^F^residues in allowed regions; ^G^residues in generally allowed regions; ^H^residues in disallowed regions; ^I^ *G*-factor score of the dihedral bonds; ^J^ *G*-factor score of the covalent bonds; ^K^overall *G*-factor score; ^L^root mean square deviation between C*α* Der f 25 structure and 2I9E template.

**Table 2:** Predicted B and T cell epitopes of Der f 25 allergen.

Peptide	Type of epitope	Position	Sequence
P1	B	11–18	*W * **K**M*N * **G** *N * **K**T
P2	B	30–35	**G** *PL * **D**S*N*
P3	B	71–77	**G** *AF*T**G** **E** *I*
P4	B	99–107	*NV* *F * **G** **E**S**D**Q*L*
P5	B	132–138	**E** **R** **E** *A * **G** **K**T
P6	B	173–18	**K**T*A*S*P*QQ*A*Q**E** *V*HQ**K** *L*
P7	B	193–197	**E** *NV*S*P*
P8	B	211–224	*V*T*ANNA * **K** **E** *LA*SQ*A * **D**
P9	T	26–34	*FL * **K** *N * **G** *PL * **D**S
P10	T	38–54	*VV* *V * **G** *VP* *AI* *YL*M*L*C**K** *NIL*
P11	T	66–74	*Y * **K** *V * **D** **K** **G** *AF*T**G** **E** *I*
P12	T	142–151	*VF * **R**QTQ*VI*S**K**
P13	T	239–247	*FV*Q*IVNA * **R**Q

Charged residues are shown in a bold font; hydrophobic residues are depicted in italic.

**Table 3:** The results of B and T cell epitopes predictions.

	Tools	Location of the prediction results
B cell epitope prediction	DNAStar protean	11–19, 28–35, 68–77, 95–111, 130–139, 173–187, 193–197, 211–225.
	BPAP	20–26, 32–52, 54–70, 86–93, 138–168, 175–209, 218–242.
	BepiPred	11–18, 30–34, 71–78, 99–107, 132–138, 168–183, 194–199, 210–224.

T cell epitope prediction (HLA-DR)	DRB1*01:01	5–13, 11–19, 10–18, 13–21, 24–32, 26–34, 39–47, 46–54, 50–58, 53–61, 57–65, 58–66, 60–68, 66–74, 73–81, 82–90, 89–97, 90–98, 104–112, 107–115, 114–122, 119–127, 122–130, 143–151, 142–150, 156–164, 157–165, 161–169, 163–171, 167–175, 187–195, 190–198, 191–199, 195–203, 197–205, 203–211, 205–213, 227–235, 228–236, 232–240, 235–243, 239–247.
	DRB3*01:01	53–61, 66–74, 81–89, 191–199.
	DRB4*01:01	26–34, 38–46, 46–54, 48–56, 50–58, 107–115, 142–150, 158–166, 160–168, 238–246, 239–247.
	DRB5*01:01	5–13, 11–19, 13–21, 20–28, 24–32, 26–34, 39–47, 46–54, 59–67, 66–74, 73–81, 89–97, 90–98, 143–151, 146–154, 150–158, 163–171, 165–173, 166–174, 203–211, 228–236, 238–246, 239–247.

T cell epitope prediction (HLA-DQ)	HLA-DQA10101-DQB10501	153–161, 154–162, 159–167, 160–168.
	HLA-DQA10102-DQB10602	33–41, 40–48, 42–50, 57–65, 77–85, 117–125, 154–162, 192–200, 206–214, 213–221, 230–238, 237–245.
	HLA-DQA10501-DQB10201	19–27, 39–47, 37–45, 82–90, 100–108, 112–120, 122–130, 126–134, 158–166, 195–203, 220–228.
	HLA-DQA10501-DQB10301	6–14, 34–42, 38–46, 39–47, 58–66, 66–74, 69–77, 72–80, 74–82, 77–85, 82–90, 85–93, 107–115, 117–125, 121–129, 133–141, 161–169, 166–174, 167–175, 169–177, 172–180, 192–200, 195–203, 203–211, 205–213, 206–214, 207–215, 213–221, 215–233, 228–236, 230–238, 232–240, 237–245.
